# #TraumaTok—TikTok Videos Relating to Trauma: Content Analysis

**DOI:** 10.2196/49761

**Published:** 2024-11-07

**Authors:** Alix Woolard, Rigel Paciente, Emily Munro, Nicole Wickens, Gabriella Wells, Daniel Ta, Joelie Mandzufas, Karen Lombardi

**Affiliations:** 1 The Kids Research Institute Australia Nedlands Australia; 2 The University of Western Australia Crawley Australia; 3 Edith Cowan University Joondalup Australia

**Keywords:** trauma, traumatic events, traumatic stress, TikTok, public health, social media, content analysis

## Abstract

**Background:**

Experiencing a traumatic event can significantly impact mental and emotional well-being. Social media platforms offer spaces for sharing stories, seeking support, and accessing psychoeducation. TikTok (ByteDance), a rapidly growing social media platform, is increasingly used for advice, validation, and information, although the content of this requires further study. Research is particularly needed to better understand TikTok content relating to trauma and the potential implications for young viewers, considering the distressing nature of the subject and the possibility of users experiencing vicarious trauma through exposure to these videos.

**Objective:**

This study aims to explore the content of trauma-related videos on TikTok, focusing on hashtags related to trauma. Specifically, this study analyzes how TikTok videos present information, advice, stories, and support relating to trauma.

**Methods:**

A quantitative cross-sectional descriptive content analysis was performed on TikTok in December 2022. A total of 5 hashtags related to trauma were selected: #trauma, #traumatized, #traumatok, #traumatic, and #traumabond, with the top 50 videos from each hashtag analyzed (total N=250 videos). A standardized codebook was developed inductively to analyze the content of the videos, while an existing generic codebook was used to collect the video features (eg, age of people in the video) and metadata (likes, comments, and shares) for each video.

**Results:**

A total of 2 major content themes were identified, which were instructional videos (54/250, 21.6%) and videos disclosing personal stories (168/250, 67.3%). The videos garnered significant engagement, with a total of 296.6 million likes, 2.3 million comments, and 4.6 million shares, indicating that users find this content engaging and useful. Alarmingly, only 3.7% (9/250) of videos included a trigger warning, despite many featuring highly distressing stories that young people and those with trauma may be exposed to.

**Conclusions:**

The study highlights the potential risks of vicarious trauma due to trauma dumping without trigger warnings on TikTok, and the need for further research to assess the accuracy of advice and information in these videos. However, it also underscores the platform’s potential to foster social connections, provide validation, and reduce stigma around mental health issues. Public health professionals should leverage social media to disseminate accurate mental health information, while promoting user education and content moderation to mitigate potential harms. People often use social media, such as TikTok to share advice, stories, and support around mental health, including their experiences with trauma. Out of 250 videos, most were either giving advice (54/250, 21.6%) or sharing personal experiences (168/250, 67.3%). The study found many videos lacked warnings about upsetting content, which could potentially harm young viewers or people suffering from trauma. While TikTok can help people feel connected and reduce the stigma around mental health, it is important to seek support from professionals when needed.

## Introduction

Approximately 70% of the global population will experience a traumatic event in their lifetime, and the prevalence of posttraumatic stress disorder (PTSD) is about 3.9% [1]. Trauma can detrimentally affect a person’s mental and physical health and social and emotional well-being, therefore addressing trauma recovery is a top priority for health professionals [2]. Studies demonstrate social support and narrative therapy (ie, telling one’s story) are imperative to trauma recovery [3]. Psychoeducation is also a useful therapeutic tool for individuals recovering from trauma [3].

A social media space may be frequented by help-seeking individuals and health professionals alike, as it can be a place to share stories, a tool to either seek or provide support, or a source of psychoeducation for individuals impacted by trauma. Individuals who have experienced trauma can view such social media help-seeking as a positive experience that aids in their recovery [4]. Young people view the accessible health information on social media as simple and accurate, meaning it can be an effective medium for education [5,6]. In recent years, TikTok (ByteDance) has become not only the fastest growing social media platform [7], but it is also an increasingly popular source of advice, validation, and information [8]. The video-based platform is widely used to express both personal experiences of users and also disseminate mental health education and information [9].

A previous study exploring mental health information and social support on TikTok [9] described the widespread engagement within the user base and highlighted how the platform can be a useful space for support and validation. To date, there have been no studies to explore the use of TikTok by individuals impacted by trauma specifically, which is surprising given the widespread nature of traumatic events. This paper aimed to address this gap by exploring the content of trauma-related videos through hashtags relating to trauma on TikTok.

## Methods

### Overview

The method for investigation of trauma-related videos on TikTok used the protocol as outlined by Mandzufas et al [10]. The hashtags with the most views relating to trauma on TikTok were selected for investigation: #trauma (14.6 billion views), #traumatized (1.8 billion views), #traumatok (2.6 billion views), #traumatic (768.9 million views), and #traumabond (581.5 million views). The first 50 videos appearing in each hashtag were selected for sampling and downloaded for analysis on December 8, 2022. Videos not in English or not relevant to trauma were excluded, and the next eligible video in order of appearance was selected instead (total N=250 videos). A total of 5 videos were excluded from the final analysis as they were duplicated videos, and they were removed from the hashtags #traumatok and #traumatic, as they had already been coded within #trauma.

The development of codebooks for the content analysis used a combined deductive and inductive approach with the addition of topic-specific variables as trialed and refined by the research team (Multimedia Appendix 1). To test the interrater reliability of the codebook, AW (primary coder) and EM (secondary coder) independently coded 5 videos randomly sampled from each hashtag (n=25). Agreement between the 2 coders was determined, with 82% agreement between the 2 coders in the first instance. Once the variables and associated definitions were finalized, a web-based survey to code the videos was created and managed in REDCap (Research Electronic Data Capture; Vanderbilt University), a secure web-based software platform hosted at the Telethon Kids Institute. All videos (N=250) were coded for trauma-related variables and captured engagement metadata (comments, likes, and shares). Descriptive statistics of the data were analyzed in Microsoft Excel.

### Ethical Considerations

This study received ethical exemption from the University of Western Australia Human Research Ethics Committee (reference number 2022/ET000058). All individual user information and posts and screenshots were deidentified in the paper and all supporting documentation.

## Results

Overall, 245 videos contributed to a total of 296.6 million likes (mean 1.2 million, SD 1.0 million), 2.3 million (mean 9453.5, SD 11,360.6) comments, and 4.6 million (mean 18,817.9, SD 27,034.3) shares. Table 1 shows the content across the 5 hashtags. Most videos were created by adults (191/245, 78% videos), and a few videos were posted by children and adolescents (20/245, 8.2% videos). Furthermore, most videos were posted by female users (162/245, 66.1% videos) compared with male users (47/245, 19.2% videos).

Overall, there were 2 major content themes in the most popular videos relating to trauma, which are instructional videos (52/245, 21.6% videos) and those that allowed users to disclose their personal stories (165/245, 67.3% videos). Although most instructional videos were made by self-titled “experts” (39/245, 15.9% videos, full list mentioned in Table 1), 18% (44/245) of the videos also promoted products, the most prominent being “mental health coaching” (n=19, full list mentioned in Table 2). Furthermore, many of the experts did not disclose a qualification.

These products and services were stated within the video as well as user’s biographies. The incongruence between the total count in the table versus the total number of videos that mentioned a product or service is due to some videos promoting multiple products or services. The percentage value is also based on the proportion of the total number of videos (N=245).

Most videos featured individuals sharing their stories and disclosing experiences of traumatic stress or PTSD (165/245, 67.3%, and 134/245, 55.1%, respectively). The most reported trauma was child maltreatment, and a smaller number of videos involved the speaker talking about the symptoms of PTSD that they experience. Other types of traumas found in the videos are listed in Table 3, and other symptoms of PTSD mentioned in the videos are listed in Table 4. Almost half (115/245, 47%) of these videos were humorous, which aligns with research that shows humor can be a powerful coping mechanism for dealing with traumatic stress [11,12]. Alarmingly, only 3.7% (9/245) of these videos had a trigger warning in some form, although many videos described highly distressing stories involving traumatic events (ie, trauma dumping). This result is concerning, given the large proportion of young users who are at high risk for vicarious traumatization (ie, second-hand trauma) after hearing distressing stories on social media without warning [13]. More in-depth hashtag breakdowns of each variable listed above can be found in Multimedia Appendix 1.

**Table 1:** Frequency of stated qualifications of self-titled experts in the current study in TikTok videos using hashtags relating to trauma. These titles were acquired from the posters’ biography sections. The percentage value is also based on the proportion of the total number of videos (N=245).

Stated self-titled qualifications	Values, n (%)
Adult and pediatric psychiatrist	1 (0.4)
Anxiety healer	2 (0.8)
Author, licensed therapist, trauma coach, influencer, and socially conscious entrepreneur	4 (1.6)
Certified trauma recovery coach	2 (0.8)
Clinical psychologist	1 (0.4)
Full-time healer	1 (0.4)
I help battered women heal from the past	1 (0.4)
Life coach	1 (0.4)
Medic	1 (0.4)
Mental health coach	1 (0.4)
Narcissistic abuse recovery coach	2 (0.8)
Neuropsychologist	1 (0.4)
Physician	1 (0.4)
“Psychology/health/biology”	1 (0.4)
Public speaker	1 (0.4)
Relationship coach	2 (0.8)
Science educator	2 (0.8)
Somatic and trauma practitioner	1 (0.4)
Therapist	2 (0.8)
Therapist in training	1 (0.4)
Trauma bond and abuse recovery coach	4 (1.6)
Trauma-informed and certified coach	1 (0.4)
Trauma speaker	4 (1.6)
Trauma therapist	1 (0.4)
Total	39 (15.9)

**Table 2:** Frequency of products and services offered by users in the current study, with self-titled qualifications in TikTok videos using hashtags relating to trauma.

Type of product			Values, n (%)
Healing services			2 (0.8)
Book			6 (2.4)
EFT^a^ tapping			2 (0.8)
Mental health, n (%)			
	Coaching	19 (7.8)	
	Online course	1 (0.4)	
	Merchandise	3 (1.2)	
Music			2 (0.8)
Performance coach training			1 (0.4)
Podcast			7 (2.9)
Support groups			1 (0.4)
Therapy service			3 (1.2)
Toastmasters			1 (0.4)
Trauma bond recovery course			1 (0.4)
Total			49 (20)

^a^EFT: emotional freedom techniques.

**Table 3:** Different types of traumatic experiences as disclosed by users in this study, investigating TikTok videos with hashtags relating to trauma. The percentage is based on the total number of videos (N=245).

Types of traumas mentioned	Values, n (%)
Child maltreatment	75 (30.6)
Community violence	1 (0.4)
Death	18 (7.3)
Familial substance abuse	3 (1.2)
Family domestic violence	24 (9.8)
Medical trauma	14 (5.7)
Natural disaster	2 (0.8)
Not specified	13 (5.3)
Other	6 (2.4)
Physical assault	6 (2.4)
Sexual assault	13 (5.3)
Vicarious trauma	2 (0.8)
Total	177 (72.2)

**Table 4:** User-mentioned posttraumatic stress disorder symptoms in this study within TikTok videos with hashtags relating to trauma. The percentage is based on the total number of videos (N=245).

Symptoms of PTSD^a^	Values, n (%)
Intrusion	21 (8.6)
Avoidance	16 (6.5)
Negative alterations in mood/thought	30 (12.2)
Arousal and reactivity	19 (7.8)
Total	86 (35.1)

^a^PTSD: posttraumatic stress disorder.

## Discussion

This paper aimed to explore the use of TikTok by individuals impacted by trauma by exploring the content of trauma-related videos. A total of 2 major content themes were identified in the most popular videos relating to trauma which are instructional videos and those that allowed users to disclose their personal stories.

Sharing experiences on social media can foster a sense of connectedness and validation. Thus, TikTok may be a means of receiving validation from others who have experienced similar adversity and can help reduce the stigma that surrounds mental health issues and trauma. If users choose to disclose their trauma on TikTok, it is important to do so with caution, with trigger warnings, and to seek additional support from professionals as needed.

The use of TikTok by those who have experienced trauma and poor mental health highlights the urgent need for better mental health literacy and content moderation on social media platforms. To complement this, public health professionals could consider leveraging these platforms to provide accurate and supportive mental health resources, ensuring users receive reliable information. In addition, educational campaigns could be developed to inform users about the risks of vicarious trauma as discussed and the importance of seeking professional help when dealing with traumatic experiences. Crucially, social media platforms should also implement and enforce guidelines for trigger warnings to protect vulnerable users from exposure to potentially distressing content.

The results of this study have several public health implications. First, given there were very few trigger warnings and the content of videos relating to trauma can be triggering for young people, the videos relating to trauma may contribute to vicarious traumatization. Furthermore, while TikTok can be a space that provides a sense of community for its users, it is not a platform designed to provide mental health support. We do not yet know the impact of disclosing trauma on a public social media platform; however, individuals impacted by trauma should be seeking mental health support from trained professionals.

There are several limitations to this study. First, the analysis was confined to videos in English, potentially excluding relevant content in other languages and from other cultures. Second, the selection process of the top 50 videos per hashtag may not capture the full spectrum of trauma-related content on TikTok, nor does it accurately represent the content a user may be exposed to when they are not specifically searching for trauma content. Importantly, this was a descriptive study conducted at a single point in time, which limits the ability to infer causality or long-term effects. The cross-sectional design means we only captured a snapshot of the content available during the study period, and the rapidly changing nature of social media content means that the videos analyzed may not be representative of all trauma-related content on TikTok over time. Future research should address these gaps by including a more diverse sample and conducting longitudinal studies to observe changes over time. In addition, it was beyond the scope of our research to investigate user experiences or the validity and accuracy of expertise conveyed in the videos. Future studies should endeavor to investigate the impact that viewing and disclosing trauma-related videos on TikTok has on its users and whether accurate information and advice are being presented in trauma-related videos on TikTok.

By fostering a safer environment on social media, users can harness the benefits of social media while mitigating its potential harms, ultimately contributing to better mental health outcomes for all users. This study underscores the need for a multifaceted approach to addressing trauma on social media, combining user education, platform responsibility, and professional support to create a healthier digital landscape.

## Appendix

Multimedia Appendix 1 Codebook used to analyze content of TikTok videos.

## Abbreviations

PTSD: posttraumatic stress disorder
REDCap: Research Electronic Data Capture

## Data Availability

The datasets generated during this study are not publicly available due to confidentiality; however, our analyzed data are available from the corresponding author on reasonable request.
