# Supplementary material for: #TraumaTok—TikTok Videos Relating to Trauma: Content Analysis
Source: JMIR Form Res. 2024 Nov 7;8:e49761. doi: 10.2196/49761 (PMC11582486; doi:10.2196/49761)
Supplement: Multimedia Appendix 1 [file formative_v8i1e49761_app1.docx]

1. Variable
2. Coder name
3. Date coded
4. URL
5. Age of main person in the video. If more than 1 main person, write all that apply.
6. Is a child present in the video? (i.e., a child must be visible)
7. If yes, age of child
8. What is the gender presentation of the main person in the video? (If more than 1 main person, write all that apply from: Male, Female, Non-binary, Unclear, Other not listed)
9. Setting (i.e., location)
10. Instructional/Educational?
11. Are they giving advice?
12. Does the speaker/content creator claim to be qualified? (i.e., claims to be accredited/qualified professional or evidence-based organisation)
    1. If yes, what is their qualification? Please write:
13. Is the content creator citing evidence-based techniques?
    1. If yes, please specify:
14. Are interactions encouraged? (i.e., likes/tags/comments)
15. Duet?
16. Stitching?
17. Branded content? (Intentional ad)
18. Are they promoting a product/service? (e.g., book, podcast, counselling service)
    1. If yes, please specify:
19. Warning or disclaimer present? (Sometimes written as TW - trigger warning)
20. Is the video obviously scripted/rehearsed? (Person has a plan or idea of how the video would look, vs video is captured in the moment/candid)
21. Type of trauma mentioned.
    1. Child maltreatment (emotional abuse)
    2. Child maltreatment (physical abuse)
    3. Child maltreatment (sexual abuse)
    4. Child maltreatment (neglect)
    5. Emotional abuse (in adulthood)
    6. Sexual abuse (in adulthood)
    7. Physical abuse (in adulthood)
    8. Financial abuse (in adulthood)
    9. Sexual assault (in adulthood, i.e. single incident)
    10. Physical assault (in adulthood)
    11. Verbal assault (in adulthood)
    12. Bullying
    13. Death
    14. Medical trauma (e.g. accident, illness)
    15. Vicarious trauma (i.e. watching or hearing someone else’s trauma)
    16. Refugee
    17. War
    18. Natural disaster
    19. Community Violence
    20. Family Domestic Violence (FDV)
    21. Familial substance abuse
    22. Familial incarceration
    23. Intergenerational trauma (i.e. parents or grandparents experienced trauma and are passing on behaviours)
    24. Not specified
    25. Unsure
    26. Other
22. Do they mention/list symptoms? Yes/No
    1. If yes, what do they mention/list?
       1. Intrusion
          1. Memories
          2. Nightmares
          3. Flashbacks
          4. Emotional distress
       2. Avoidance
          1. Events/situations
          2. Thoughts/feelings
       3. Negative alterations in mood/thoughts
          1. Memory loss
          2. Negative thoughts
          3. Blame
          4. Isolation
       4. Negative affect (i.e. emotion)
          1. Decreased interest in activities
          2. Difficulty feeling positive feelings (lack of positive feeling rather than feeling bad)
       5. Arousal and reactivity
          1. Irritability/aggression
          2. Hypervigilance (being more aware of environment)
          3. Heightened startle reaction
          4. Risky behaviour (i.e. sex, drugs, alcohol)
          5. Sleep problems
          6. Difficulty concentrating
23. Is personal experience referenced or insinuated? (Captures personal story)
24. Do they mention perpetrators (refers to illegal behaviours)?
25. Is the creator suggesting a diagnosis based on symptoms or an event? (e.g., PTSD, Anxiety, Depression)
26. Are they trauma dumping?
27. Are comments disabled? (i.e., turned off)
